# Genetic targeting or pharmacological inhibition of galectin-3 dampens microglia reactivity and delays retinal degeneration

**DOI:** 10.1186/s12974-022-02589-6

**Published:** 2022-09-17

**Authors:** Mona Tabel, Anne Wolf, Manon Szczepan, Heping Xu, Herbert Jägle, Christoph Moehle, Mei Chen, Thomas Langmann

**Affiliations:** 1 Laboratory for Experimental Immunology of the Eye, Department of Ophthalmology, University of Cologne, Faculty of Medicine and University Hospital Cologne, Cologne, Germany; 2 Wellcome-Wolfson Institute for Experimental Medicine, School of Medicine, Dentistry and Biomedical Sciences, Queen’s University Belfast, Belfast, UK; 3 Department of Ophthalmology, University Eye Clinic Regensburg, Regensburg, Germany; 4 Center of Excellence for Fluorescent Bioanalytics, University of Regensburg, Regensburg, Germany; 5 Centre for Molecular Medicine Cologne (CMMC), University of Cologne, Cologne, Germany

**Keywords:** Galectin-3 deficiency, Galectin-3 inhibition, TD139, Microglia, Retinal degeneration, Light damage

## Abstract

**Background:**

Dysfunctional humoral and cellular innate immunity are key components in the development and progression of age-related macular degeneration (AMD). Specifically, chronically activated microglia and their disturbed regulatory system contribute to retinal degeneration. Galectin-3, a β-galactose binding protein, is a potent driver of macrophage and microglia activation and has been implicated in neuroinflammation, including neurodegenerative diseases of the brain. Here, we hypothesized that genetic deficiency of galectin-3 or its modulation via TD139 dampens mononuclear phagocyte reactivity and delays retinal degeneration.

**Methods:**

Galectin-3 expression in AMD patients was analyzed by immunohistochemical stainings. Galectin-3 knockout and BALB/cJ mice were exposed to white bright light with an intensity of 15,000 lux for 1 h and Cx3cr1^GFP/+^ mice to focal blue light of 50,000 lux for 10 min. BALB/cJ and Cx3cr1^GFP/+^ mice received intraperitoneal injections of 15 mg/kg TD139 or vehicle for five consecutive days, starting one day prior to light exposure. The effects of galectin-3 deficiency or inhibition on microglia were analyzed by immunohistochemical stainings and in situ hybridization of retinal sections and flat mounts. Pro-inflammatory cytokine levels in the retina and retinal pigment epithelium (RPE) were quantified by qRT-PCR and transcriptomic changes were analyzed by RNA-sequencing. Retinal thickness and structure were evaluated by optical coherence tomography.

**Results:**

We found that galectin-3 expression was strongly upregulated in reactive retinal mononuclear phagocytes of AMD patients and in the two related mouse models of light-induced retinal degeneration. The experimental in vivo data further showed that specific targeting of galectin-3 by genetic knockout or administration of the small-molecule inhibitor TD139 reduced microglia reactivity and delayed retinal damage in both light damage conditions.

**Conclusion:**

This study defines galectin-3 as a potent driver of retinal degeneration and highlights the protein as a drug target for ocular immunomodulatory therapies.

**Supplementary Information:**

The online version contains supplementary material available at 10.1186/s12974-022-02589-6.

## Background

Age-related macular degeneration (AMD) is a relatively common and progressive neurodegenerative disease of the elderly and a leading cause of vision loss in the western world [1]. Early AMD is characterized by lipid-rich deposits, so called soft drusen, between the outer retina and the retinal pigment epithelium (RPE) or underneath the RPE [2, 3]. Advanced stages can be classified as either dry or wet AMD, characterized by geographic atrophy (GA) and choroidal neovascularization, respectively. While aberrant vessel growth in wet AMD patients can be treated with repeated intravitreal injections of anti-VEGF drugs, there is currently no approved treatment option available for GA, accounting for 80–90% of all AMD cases [4, 5]. Although the etiology of AMD is not completely understood, dysregulation of ocular innate immunity has been identified as a relevant pathomechanism in the development and progression of AMD [6]. Retinal microglia, the tissue resident immune cells, play pivotal roles in innate immune responses and regulation of tissue integrity. Microglia reactivity is triggered by a variety of local disease-related factors including the complement system, modified cell surfaces and cell debris [7–9]. While a short period of microglia activation supports tissue homeostasis, chronic microglia reactivity represents a driving force for retinal cell death and disease manifestation [10–12]. Therefore, selective modulation of microglia reactivity is considered as a potential treatment option for immune‐related retinal diseases.

Recently, we have shown that long-chain polysialic acids form a protective layer on retinal cells and that their binding to microglial Siglec11 receptors is strongly anti-inflammatory, indicating a crucial role for glycobiology in retinal immunity [13]. Galectin-3, a member of the β-galactoside-binding lectin family, is considered as a potent mediator of brain microgliosis and inflammation [14–17]. In its secreted form, galectin-3 is a potent ligand for Toll-like receptor 4 (TLR4) signaling [15]. Besides its role in inflammation, it is also involved in cell adhesion, phagocytosis, and cell death [16, 18–20]. Increased galectin-3 expression in human brain samples has been documented in the pathology of different neurodegenerative diseases including Alzheimer’s disease (AD) [21, 22] and Huntington’s disease (HD) [23]. In the eye, galectin 3 has been identified in a distinct transcriptomic cluster associated with light-damage triggered subretinal microglia [24]. Interestingly, loss of galectin-3 attenuated the microglia-associated immune responses, thus improving disease outcome in mouse models of ischemic brain injury, AD and HD [16, 21, 23]. Furthermore, pharmacological inhibition of galectin-3 exerted potent immunomodulatory properties in a mouse model of Parkinson´s disease (PD) [25].

In the present study, we demonstrate that galectin-3 is strongly upregulated in retinas of dry AMD patients and in two different corresponding mouse models. This increased galectin-3 expression is prominently found in reactive microglia. Using the light-induced retinal degeneration model, an established mouse model to study key aspects of dry AMD [26], we show that genetic deficiency and pharmacological inhibition of galectin-3 both can diminish microglia reactivity and delay light-induced retinal degeneration. Collectively, our findings reveal that increased galectin-3 in microglia contributes to inflammation-related disease progression, thus highlighting the protein as a potential drug target for dry AMD.

## Materials and methods

### Human tissue

This study was performed in accordance with the tenets of the Declaration of Helsinki, with the Human Tissue Act (2004) and was approved by the local Ethical Review Boards of Queen’s University Belfast. The donors were 59 (healthy) and 70 (dry AMD, both eyes) years old and the post‐mortem time ranged between 12 and 15 h. For immunohistochemical analysis, eyes were fixed in formalin and embedded in paraffin wax. Six-micrometer sections were deparaffinized using xylene and 100% ethanol, washed in tap water followed by rehydration and antigen retrieval using 1 × Trilogy buffer (920P-04, Cell Marque) for 30 min at 95 °C. Then, sections were washed in hot rinse solution for 5 min at 95 °C, in fresh dH_2_O and 1 × PBS at room temperature (RT). After blocking with 10% FCS/PBS for 1 h at RT and incubation with a polyclonal goat anti-IBA-1 antibody (1:100 dilution, ab5076, Abcam) for 1 h at RT, sections were washed in 1 × PBS and incubated with donkey anti-goat AlexaFluor™ 594 (1:100 dilution, 705–585-003, Jackson Immuno Research) for 1 h at RT. Following this, sections were incubated overnight at 4 °C with a mouse monoclonal anti-galectin 3 antibody (1:100 dilution, ab2785, Abcam), washed and incubated with rabbit anti-mouse HRP antibody (1:100 dilution, P0161, Dako) for 1 h at RT. After several washing steps, the sections were incubated for 1 h at RT with a horse anti-rabbit biotinylated antibody (1:100 dilution, BA-1100, Vector) followed by an incubation with Streptavidin AlexaFluor™ 488 (1:100 dilution, S11223, Invitrogen) for 1 h at RT and embedded in fluorescence mounting medium with DAPI (Vectashield H-1200, Vector Labs) after final washing steps. Images were taken with a SP8 confocal microscopy.

### Animals

Mice were housed in individually ventilated cages (GM500, Tecniplast® Greenline) under specific pathogen-free conditions with a maximum of five adult mice per cage. Environmental cage enrichment includes bedding and nesting material, cardboard house and wooden chew sticks. Mice were maintained in an air-conditioned environment on a 12-h light–dark schedule at 22 ± 2 °C and 45–65% relative humidity. Mice were fed an irradiated standard diet (Altromin 1324: 11% fat, 24% protein and 65% carbohydrates) and had access to food and acidified water ad libitum.

B6.Cg-*Lgals3*^tm1Poi^/J mice and BALB/cJ mice, purchased from the Jackson Laboratory, were crossed to generate B6.Cg-*Lgals3*^tm1Poi^/ BALB/cJ mice (*Lgals-3* KO). For the experiments, 8- to 10-week-old BALB/cJ mice and homozygous *Lgals-3* KO mice carrying the light-sensitive RPE65^M450L^ variant and corresponding wild-type littermates of both sexes were used. Heterozygous Cx3cr1^GFP/+^ reporter mice on C57BL/6J background of both sexes were used at 13 weeks of age. All animal procedures used in this study were reviewed and approved by the governmental body responsible for animal welfare in Nordrhein-Westfalen (Landesamt für Natur, Umwelt und Verbraucherschutz Nordrhein-Westfalen: approval no. 81-02.04.2018.A303) and by the Animal Welfare and Ethical Review Board of Queen’s University Belfast under the regulation of the UK Home Office Animal (Scientific Procedures) Act 1986.

### TD139 administration

TD139 (also known as 33DFTG) was obtained by MedChem Express (HY-19940) and AOBIOUS (AOB 37,408). BALB/cJ and Cx3cr1^GFP/+^ mice received daily intraperitoneal injections of 15 mg/kg TD139 dissolved in DMSO or DMSO as a vehicle control, starting one day before light exposure. Dissolved TD139 was incubated for 10 min at 37 °C under constant agitation before use.

### Light exposure regimens

Mice were dark adapted for 16 h before light exposure. After the pupils were dilated with a topical drop of 0.5% tropicamide and 2.5% phenylephrine under dim red light, BALB/cJ and *Lgals*-3 KO mice were exposed to bright white light with an intensity of 15,000 lux for 1 h and Cx3cr1^GFP^/ + mice were exposed to focal white light with an intensity of 50,000 lux delivered by an otoscope (Karl Storz 1218AA) for 10 min. For the remaining experimental period, mice were housed under normal light conditions.

### Optical coherence tomography (OCT)

Before image acquisition, mice were anesthetized by intraperitoneal injections of xylazine (5 mg/kg, Bayer HealthCare) and ketamine (100 mg/kg body weight, Zoetis) diluted in 0.9% sodium chloride. The pupils were dilated with a topical drop of 0.5% tropicamide and 2.5% phenylephrine before imaging with the Spectralis™ HRA/OCT device (Heidelberg Engineering). One, three and four days post-light exposure, spectral-domain OCT (SD-OCT) scans were recorded to analyze the retinal structure and thickness. Retinal thickness measurements were performed using the Heidelberg Eye Explorer software with a circular ETDRS grid (circle diameters 3 mm and 6 mm) centered on the optic nerve head. The average retinal thickness for each circle diameter was calculated by the mean of the four fields, respectively. Each circle was evaluated individually, the 6-mm area documents the peripheral area, thereby excluding the central 3-mm area.

### Immunohistochemistry

One, three and four days post-light exposure mice were euthanized by cervical dislocation, the eyes enucleated and fixed in 4% formaldehyde (FA) for 3 h at RT. For cryosectioning, eyes were dehydrated in 30% sucrose and embedded in optimal cutting temperature OCT compound (Tissue-Tek®, Sakura). Ten-micrometer sections were rehydrated in 1 × PBS, blocked with dried milk solution containing 0.01% Triton X-100 and incubated with a polyclonal rabbit anti-Iba-1 antibody (1:500 dilution, 019–19,741, Wako) overnight at 4 °C. Then, sections were washed four times in 1 × PBS and incubated for 1 h with donkey anti-rabbit AlexaFluor™ 488 (1:1000 dilution, A21206, Invitrogen), donkey anti-rabbit AlexaFluor^TM^647 (1:1000 dilution, A32573, Invitrogen) or goat anti-rat Alexa Fluor 488 (1:1000 dilution, A11006, Thermo Fisher Scientific) at RT. After several washing steps, the sections were embedded in fluorescence mounting medium with DAPI (Fluoromount-G™ with DAPI, Invitrogen).

Retinal and RPE/choroidal flat mounts were stained with a polyclonal rabbit anti-Iba-1 antibody (1:500 dilution, 019-19741, Wako) as previously described [27]. Here, flat mounts were incubated for 1 h with donkey anti-rabbit AlexaFluor™ 488 (1:1000 dilution, A21206, Invitrogen) or donkey anti-rabbit AlexaFluor™ 647 (1:1000 dilution, A31573, Invitrogen). After three washing steps in PBST-X (0.3% Triton X-100 in PBS), flat mounts were mounted on a microscope slide and embedded with fluorescence mounting medium (Vectashied HardSet H-1400, Vector Labs). Images from cryosections and flat mounts were taken with a Zeiss Imager.M2 equipped with an ApoTome.2. The Iba-1 positive area was analyzed by the threshold tool of Image J (v2.1.0/1.53c) within a specific area as defined by Roj Manager.

For cone arrestin staining of retinal flat mounts, Cx3cr1^GFP/+^mice were euthanized by CO_2_ inhalation (10.5L/min -300 s). Eyes were fixed in 2% FA for 2 h, dissected and permeabilized in 1 × PBS containing 1% Triton X-100 for 4 h followed by an overnight incubation with a polyclonal rabbit anti-cone arrestin antibody (1:150 dilution, Merck Millipore, AB152282) at 4 °C. After three washing steps for 30 min with 1 × PBS, retinal flat mounts were incubated with donkey anti-rabbit AlexaFluor™ 594 (1:150 dilution, 711–585-154, Jackson Immuno Research), mounted on a microscope slide and embedded with fluorescence mounting medium (Vectashield H-1000, Vector Labs). Here, images were taken with a SP8 confocal microscopy. Morphological changes were investigated by grid cross analysis of microglia. Therefore, we used the grid tool in ImageJ, defined a grid size of 0.001 mm^2^ and counted the number of grid crosses per microglia cell.

### RNA Scope® ISH

RNA Scope® ISH was performed with the RNAScope® Multiplex Fluorescent Reagent Kit v2 (ACD Bio-Techne), according to the manufacturer’s instructions with some modifications. In brief, fresh frozen ten-micrometer sections were baked for 1 h at 60 °C and after hybridization and washing incubated for 5 min in 1 × Target retrieval reagent at 95 °C. All washing steps and probe incubations were done for 5 min at RT. The following probes were used in this study: Mm-*Aif1* C3, ACD 319,141 and Mm-*Lgals*-3 C1, ACD 461,471. The probes were labeled with TSA® Plus Fluorophore Cyanine 3 and Fluorescein. For localization of the *Aif-1* mRNA expression, we performed a co-immunohistochemical staining with Iba-1 antibody (1:500 dilution, 019–19,741 Wako) and the secondary antibody AlexaFluor™ 647 (A-31573, Invitrogen). To analyze the expression of *Lgals-3* in infiltrating peripheral monocytes, we performed an immunohistochemical co-staining with monoclonal rat anti-CD45 antibody (1:200 dilution, 14–0451-82, Invitrogen) after in situ hybridization with the *Lgals-3* probe. From here, the same procedure was done as described in the immunohistochemistry section with the exception that all steps were performed in the dark.

The sections were embedded in fluorescence mounting medium (Dako) and images taken with a Zeiss Imager.M2 equipped with an ApoTome.2. Further analyses were performed with Image J.

### RNA isolation and reverse transcription

Total RNA was isolated from murine retina and RPE/choroidal tissue using the RNeasy Micro Kit (Qiagen) according to the manufacturer’s instructions. RNA was quantified spectrophotometrically with a NanoDrop 2000 (Thermo Scientific). First-strand cDNA was carried out from total RNA with RevertAid H Minus First-strand cDNA Synthesis kit (Thermo Scientific).

### Quantitative real-time PCR

Transcript levels were analyzed by quantitative real-time PCR performed in a LightCycler 480 II (Roche) with either probe based (LightCycler 480 Probe Master Roche) or SYBR® Green (Takyon No Rox SYBR Master Mix dTTP blue, Eurogentec) detection. Primer sequences and universal probe library (UPL) probe numbers in probe based detection were as follows: *Ccl2*, forward primer 5´-catccacgtgttggctca-3´, reverse primer 5′-gatcatcttgctggtgaatgagt-3′, Probe #62; *iNos*, forward primer 5′-ctttgccacggacgagac-3′, reverse primer 5′-tcattgtactctgagggctga-3′, Probe #13; *Il-6*, forward primer 5′-gctaccaaactggatataatcagga-3′, reverse primer 5′-ccaggtagctatggtactccagaa-3′, Probe #6; *Lgals-3*, forward primer 5′-gcctaccccagtgctcct-3′, reverse primer 5′-ggtcatagggcaccgtca-3′, Probe #18 and *Atp5b*, forward primer 5′-ggcacaatgcaggaaagg-3′, reverse primer 5′-tcagcaggcacatagatagcc-3′, Probe #77. UPL probes were purchased from Roche. For SYBR® Green detection, primer sequences were as follows: *Tspo*, forward primer 5′-ggaacaaccagcgactgc-3′, reverse primer 5′-gtacaaagtaggctcccatgaa; *Tnf-α*, forward primer 5′-ctgtagcccacgtcgtagc-3′, reverse primer 5′-ttgagatccatgccgttg-3′; *Il-1ß*, forward primer 5′-agttgacggaccccaaaag-3′, reverse primer 5′-agctggatgctctcatcagg-3′; *Cx3cr1*, forward primer 5′-cacttgcctctggtggagtc-3′, reverse primer 5′-agcagatgggaagggaactt-3′; *Trem119*, forward primer 5′-gtcactccatcccagtttcac-3′, reverse primer 5′-ggaccatgttgagctatggaa-3′; *P2ry12*, forward primer 5′-cccggagacactcatatcctt-3′, reverse primer 5′-gtcccaggggagaaggtg-3′; *Trem2*, forward primer 5′-tgggacctctccaccagtt-3′, reverse primer 5′-gtggtgttgagggcttgg-3′; *Cd68*, forward primer 5′-ctctctaaggctacaggctgct-3′, reverse primer 5′-tcacggttgcaagagaaaca-3′.

Measurements were performed in technical duplicates, *Atp5b* expression was used as most stable reference gene and the results were analyzed with the Light Cycler 480 software using delta delta CT threshold calculation for relative quantification.

### RNA extraction, library preparation and RNA sequencing

Total RNA was extracted from retinas of naïve and light-exposed mice treated with vehicle or TD139 using miRNeasy Micro Kit according to the manufacturer’s instructions. The RNA purity and integrity was determined by Agilent 2100 Bioanalyzer with the RNA 6000 Nano LabChip reagent set (Agilent, Palo Alto, CA, USA). Library preparation and RNA-seq were carried out as described in the Illumina (Stranded mRNA Prep Ligation) reference Guide, the Illumina NextSeq 2000 Sequencing System Guide (Illumina, Inc., San Diego, CA, USA), and the KAPA Library Quantification Kit—Illumina/ABI Prism (Roche Sequencing Solutions, Inc., Pleasanton, CA, USA). The libraries were bead purified twice and quantified using the KAPA Library Quantification Kit. Equimolar amounts of each library were sequenced on an Illumina NextSeq 2000 instrument controlled by the NextSeq 2000 Control Software (NCS) v1.4.0.39521, using two 50 cycles P3 Flow Cells with the dual index, single-read (SR) run parameters. Image analysis and base calling were done by the Real Time Analysis Software (RTA) v3.9.2. The resulting *.cbcl files were converted into *.fastq files with the bcl2fastq v2.20 software. Quantification of differential transcript expression was carried out with Illumina BaseSpace/RNA express software. Transcripts were considered as differentially expressed with a log2-fold change of ≥ 2 or ≤  − 2 and plotted against the false discovery rate (FDR) = -10log of q-value. Integrative analysis of genome-wide expression activities was performed with the DAVID Bioinformatics Resources and Heat maps were generated with the Morpheus software tool (Broad Institute).

### Statistical analysis

Statistical analysis was performed using GraphPad prism 7.05 Software. Data from the white light-induced retinal degeneration model were analyzed using an ordinary one-way ANOVA following Tukey’s multiple comparison test while data from the focal blue light damage were analyzed by Kruskal–Wallis test followed by Dunn’s multiple comparison. Data were presented as mean ± SEM, *P < 0.05; **P < 0.01; and ***P ≤ 0.001.

## Results

### High galectin-3 expression in retinas with geographic atrophy

**Fig. 1:**
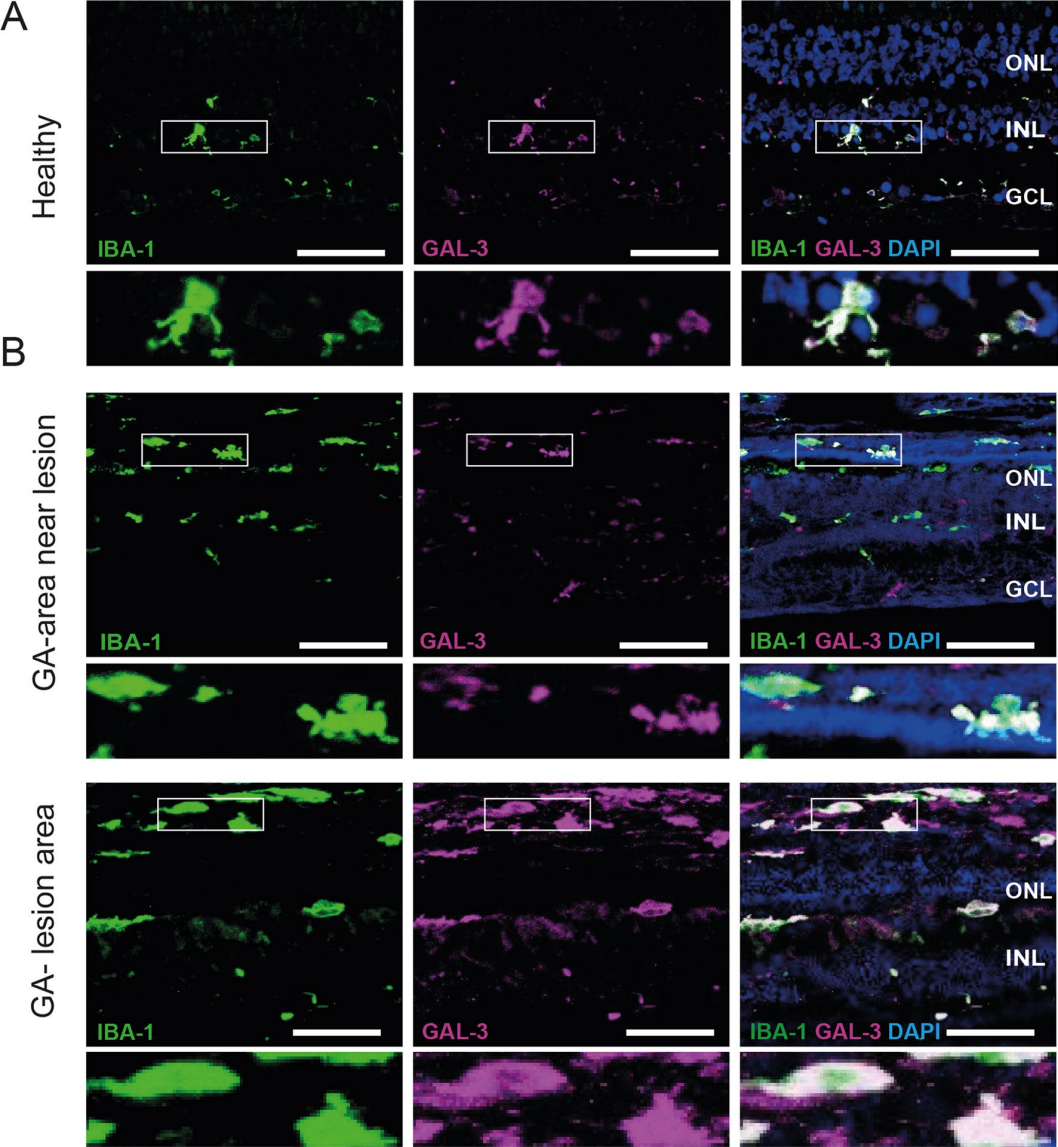
Increased galectin-3 expression in human AMD retinas. Representative images of IBA-1- and GAL-3-stained human healthy (**A**) and dry AMD retinas (**B**). Inlays show higher magnification. Scale bar: 50 µm. GA, geographic atrophy; ONL, outer nuclear layer; INL, inner nuclear layer and GCL, ganglion cell layer

High galectin-3 levels have been associated with neuroinflammation in a variety of brain diseases [21, 23, 25, 28] and proteome profiling provided first indications that the glycoprotein is also induced in human AMD [26, 29–31]. To corroborate these findings and to further define the cellular source of galectin-3 in the AMD retina, we studied its expression and localization in human dry AMD specimens. While only low levels of galectin-3 were detected in healthy human retinas, its expression was strongly increased in retinas of dry AMD patients (Fig. 1). Galectin-3 expression was mainly localized to activated microglia in the degenerating outer nuclear layer (ONL) and subretinal space in AMD patients with geographic atrophy (GA) (Fig. 1B). The expression showed a gradient towards medium levels in near lesion areas and highest levels within GA lesions (Fig. 1). Of note, a substantial galectin-3 staining was seen in subretinal lesion areas that was not completely overlapping with Iba1, indicating that other cells than microglia could produce the glycoprotein in AMD conditions.

### Galectin-3 deficiency attenuates microglia reactivity in the light-damage paradigm of retinal degeneration

**Fig. 2:**
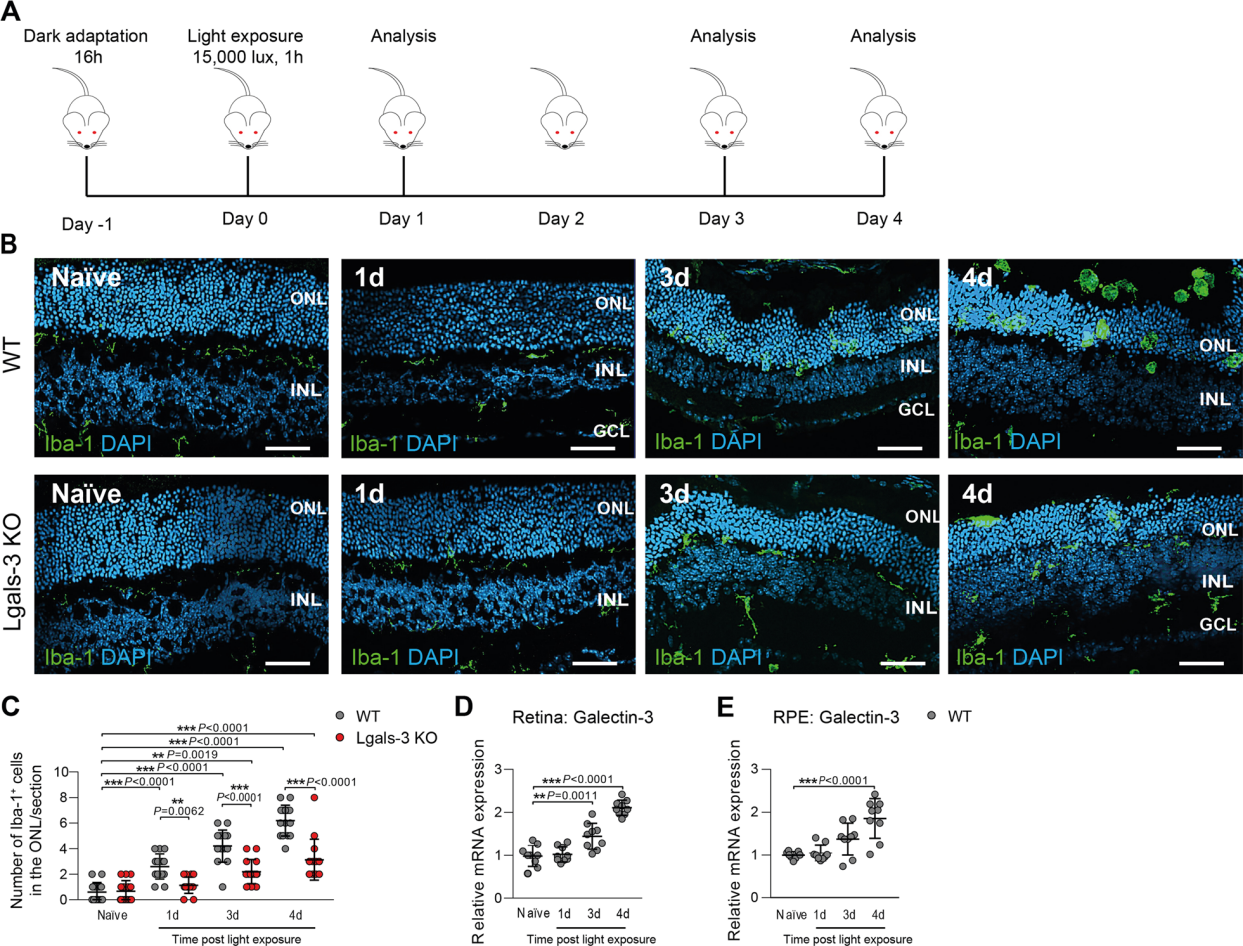
Galectin-3 deficiency reduces microglia migration in light-exposed retinas. **A** Light exposure regimen performed with *Lgals*-3 KO mice and corresponding WT littermates. **B** Representative images of Iba-1^+^ cells in naïve and light-exposed WT and *Lgals*-3 KO retinas at the indicated time points. Scale bar: 50 µm. ONL, outer nuclear layer and INL, inner nuclear layer. **C** Quantification of Iba-1^+^ cells in the ONL. n = 15 retinas from individual mice **D**,** E** Galectin-3 transcript levels in light-exposed retinas (**D**) and RPE/choroids (**E**) in WT mice calibrated to naïve tissue. *n* = 9 retinas or RPE/choroids from individual animals. Data show mean ± SEM. *P < 0.05; **P < 0.01 and ***P ≤ 0.001 by ordinary one-way ANOVA followed by Tukey’s multiple comparisons

Loss of galectin-3 has been associated with reduced neuroinflammation in mouse models of brain degeneration [16, 23, 28]. To elucidate the role of galectin-3 in retinal inflammatory diseases, we studied the light-induced retinal degeneration model mimicking key features of dry AMD including microgliosis and photoreceptor cell death [26]. Dark-adapted galectin-3 knockout mice carrying the light-sensitive RPE65^M650L^ variant (referred to as *Lgals*-3 KO) and corresponding wild type (WT) control littermates were exposed to bright white light with an intensity of 15,000 lux for 1 h (Fig. 2A). We first assessed microgliosis in galectin-3-deficient animals exposed to bright light using Iba-1-stained retinal cross-sections. While naïve control mice displayed ramified Iba-1^+^ microglia mainly in the plexiform layers, light-exposed WT retinas revealed an increased migration and accumulation of amoeboid-shaped microglia in the degeneration photoreceptor layer (ONL) and subretinal space starting one day post-light exposure (Fig. 2B, C). This severe retinal thinning associated with the accumulation of activated microglia was strongly reduced in light-exposed *Lgals*-3 KO mice (Fig. 2B, C). In wild-type animals, galectin-3 transcript levels strongly increased after light exposure in both retinal and RPE/choroidal samples, indicating that microgliosis is tightly correlated with galectin-3 mRNA expression (Fig. 2D, E).

**Fig. 3:**
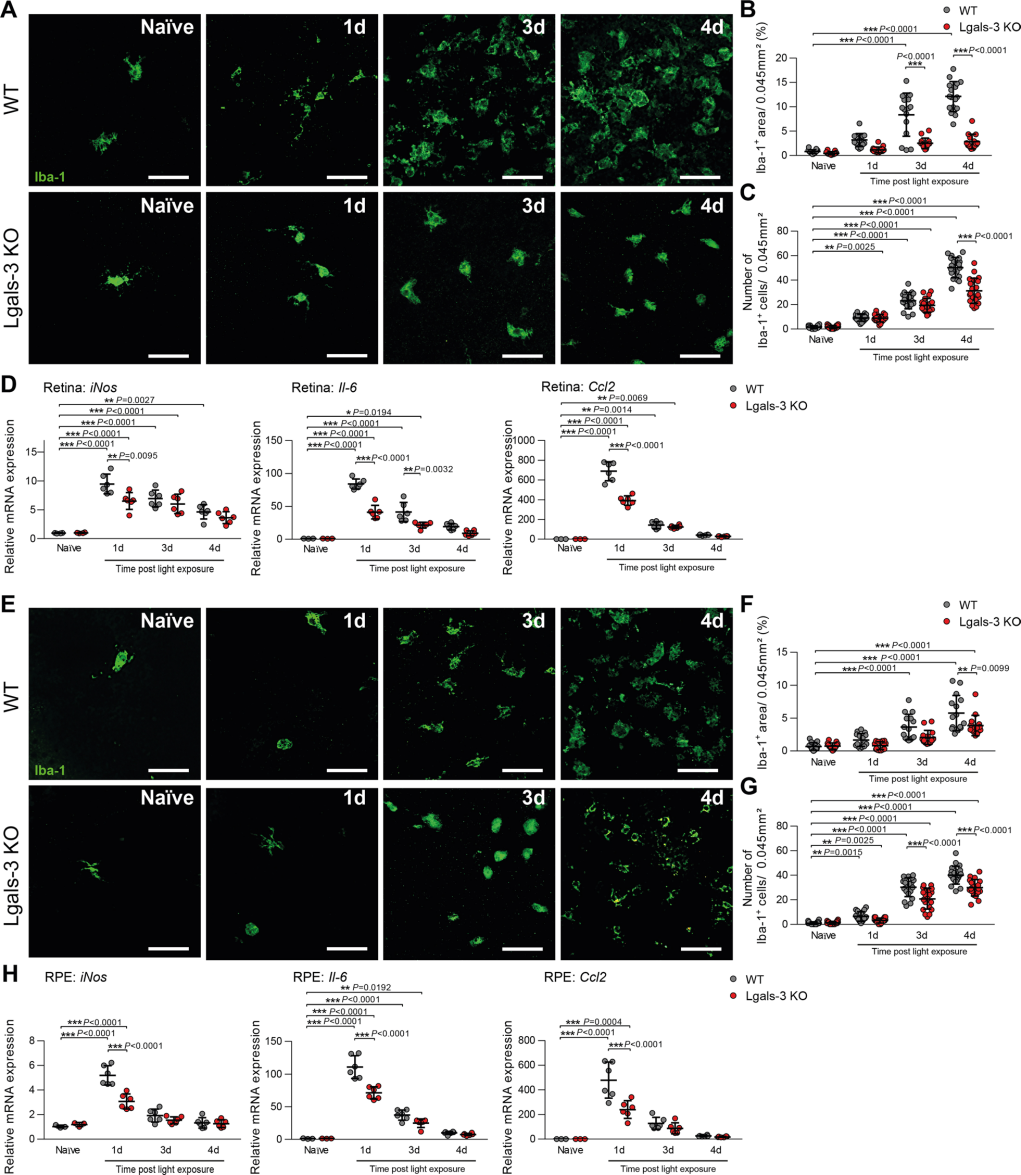
Galectin-3 deficiency dampens microglia reactivity in light-exposed mice. **A** Representative images of Iba-1^+^ cells in the subretinal space of retinal flat mounts from naïve and light-exposed WT and *Lgals*-3 KO mice. Scale bar: 50 µm. **B** Quantification of Iba-1^+^ area in the subretinal space. *n* = 15 retinas from individual mice. **C** Number of Iba-1^+^ cells in the subretinal space. *n* = 15 retinas from individual mice. **D** Relative mRNA expression of pro-inflammatory cytokines in retinas of naïve and light-exposed WT and *Lgals*-3 KO mice normalized to the reference gene Atp5b. *n* = 6 retinas except naïve *n* = 4 (*iNos*) or *n* = 3 (*Il-6,* and *Ccl2*) retinas from individual mice. **E** Representative images of Iba-1^+^ cells in RPE/choroids from naïve and light-exposed WT and *Lgals*-3 KO mice. Scale bar: 50 µm. **F** Quantification of Iba1^+^ area in RPE/choroidal flat mounts. *n* = 15 RPE/choroids from individual mice. **G** Number of Iba-1^+^ cells in the RPE/choroid. *n* = 15 RPE/choroids from individual mice. **H** Relative mRNA expression of pro-inflammatory cytokines in RPE/choroids of naïve and light-exposed WT and *Lgals*-3 KO mice normalized to the reference gene Atp5b. *n* = 6 RPE/choroids except naïve *n* = 3 RPE/choroids from individual mice. Data show mean ± SEM. *P < 0.05; **P < 0.01 and ***P ≤ 0.001 by ordinary one-way ANOVA followed by Tukey’s multiple comparisons

We next analyzed the morphological phenotypes of microglia in light-exposed WT and *Lgals*-3 KO mice using retinal flat-mount staining. In naïve retinas, only a few ramified microglia were detected in the subretinal space, whereas a significant time-dependent increase of amoeboid-shaped microglia numbers was seen in light-exposed WT retinas (Fig. 3A–C). Retinas of *Lgals*-3 KO mice revealed significantly lower microglia infiltration into the subretinal space compared to light-exposed WT retinas (Fig. 3A–C). These findings were corroborated by pro-inflammatory cytokine levels in these tissues. While high transcript levels of *iNos*, *Il-6* and *Ccl2* were detected in WT retinas with peak levels found one day after light exposure, significantly lower mRNA expression levels were detected in *Lgals*-3 KO retinas (Fig. 3D).

Next, Iba-1-stained RPE/choroidal flat mounts were analyzed to examine the effects of galectin-3 deficiency on RPE-associated mononuclear phagocyte activation. Similar to the retina, loss of galectin-3 attenuated Iba1^+^ cell infiltration in the RPE/choroid of light-exposed mice compared to light-exposed WT mice (Fig. 3E–G). Moreover, mRNA levels of *iNos*, *Il-6* and *Ccl2* were increased in the RPE/choroid after light exposure whereas loss of galectin-3 caused diminished expression (Fig. 3H). These data indicate that galectin-3 expression in light-damaged retinas coincides with mononuclear phagocyte reactivity and that loss of galectin-3 reduces their inflammatory response.

### Galectin-3 deficiency delays light-induced retinal degeneration

**Fig. 4:**
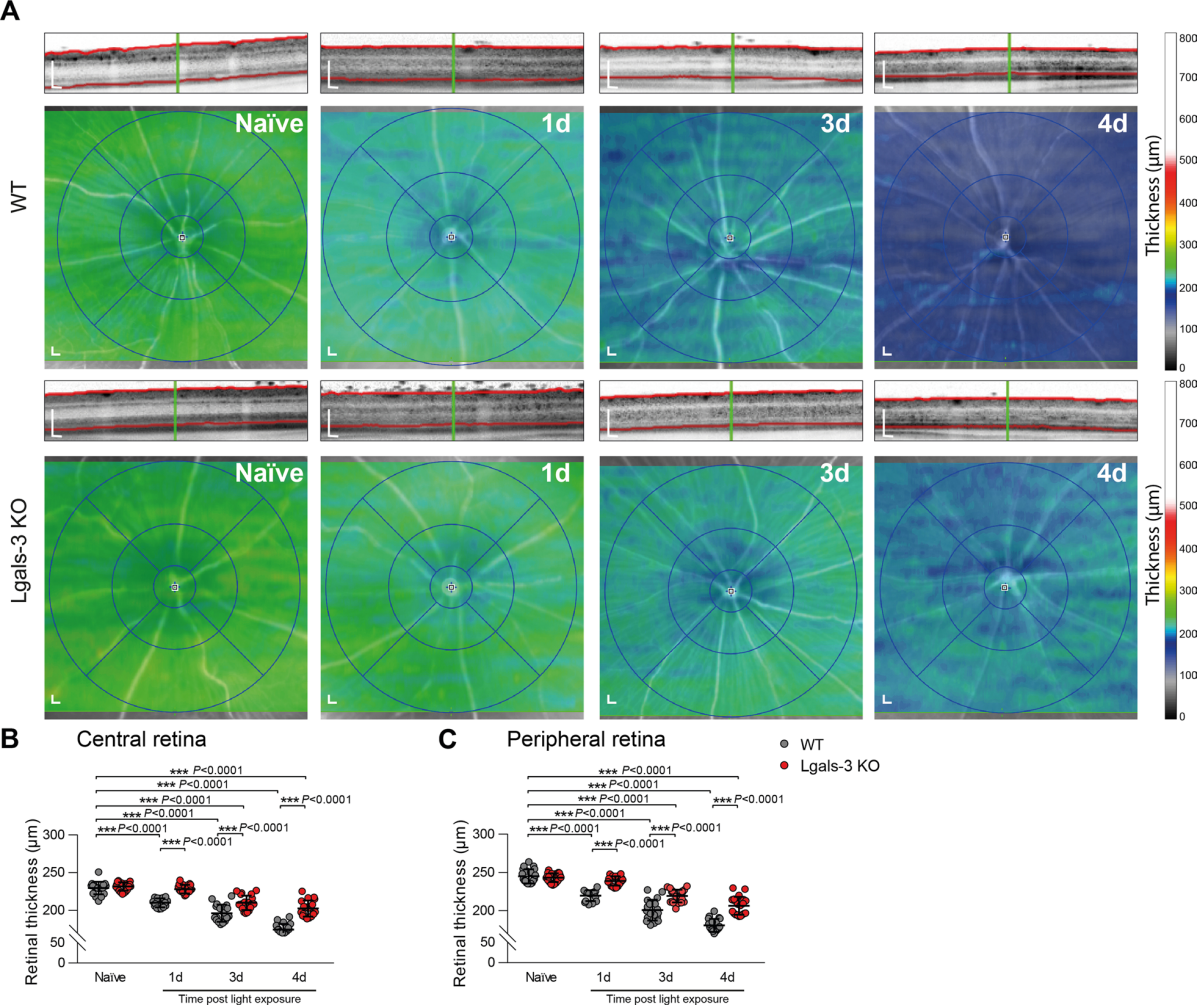
Galectin-3 deficiency delays light-induced retinal degeneration. **A** Representative SD-OCT scans and heat maps of retinal thickness from naïve and light-exposed WT and *Lgals*-3 KO mice at the indicated time points. Scale bar: 200 µm. **B**,** C** Quantification of the average thickness of the central retina (circle diameter 3 mm) (**B**) and peripheral retina (circle diameter 6 mm) (**C**). Data show mean ± SEM. Naïve *n* = 20; WT/Lgals*-*3 KO: 1d *n* = 15/19; 3d *n* = 18/20; 4d *n* = 19/20 eyes. ***P ≤ 0.001 by ordinary one-way ANOVA followed by Tukey’s multiple comparisons

We then investigated the effects of galectin-3 knockout on retinal disease progression using in vivo spectral domain optical coherence tomography (SD-OCT). SD-OCT scans of light-exposed mice revealed marked changes in the ONL reflectance compared to naïve mice (Fig. 4A). In contrast to naïve mice, light-exposed WT mice showed a strong and progressive retinal thinning, while heat maps of *Lgals*-3 KO mice revealed a clear time-dependent preservation of retinal thickness in the central area as well as periphery (Fig. 4A–C). Thus, loss of galectin-3 significantly delays retinal degeneration in this light-damage paradigm.

### Pharmacological inhibition of galectin-3 dampens microglia reactivity in light-exposed mice

**Fig. 5:**
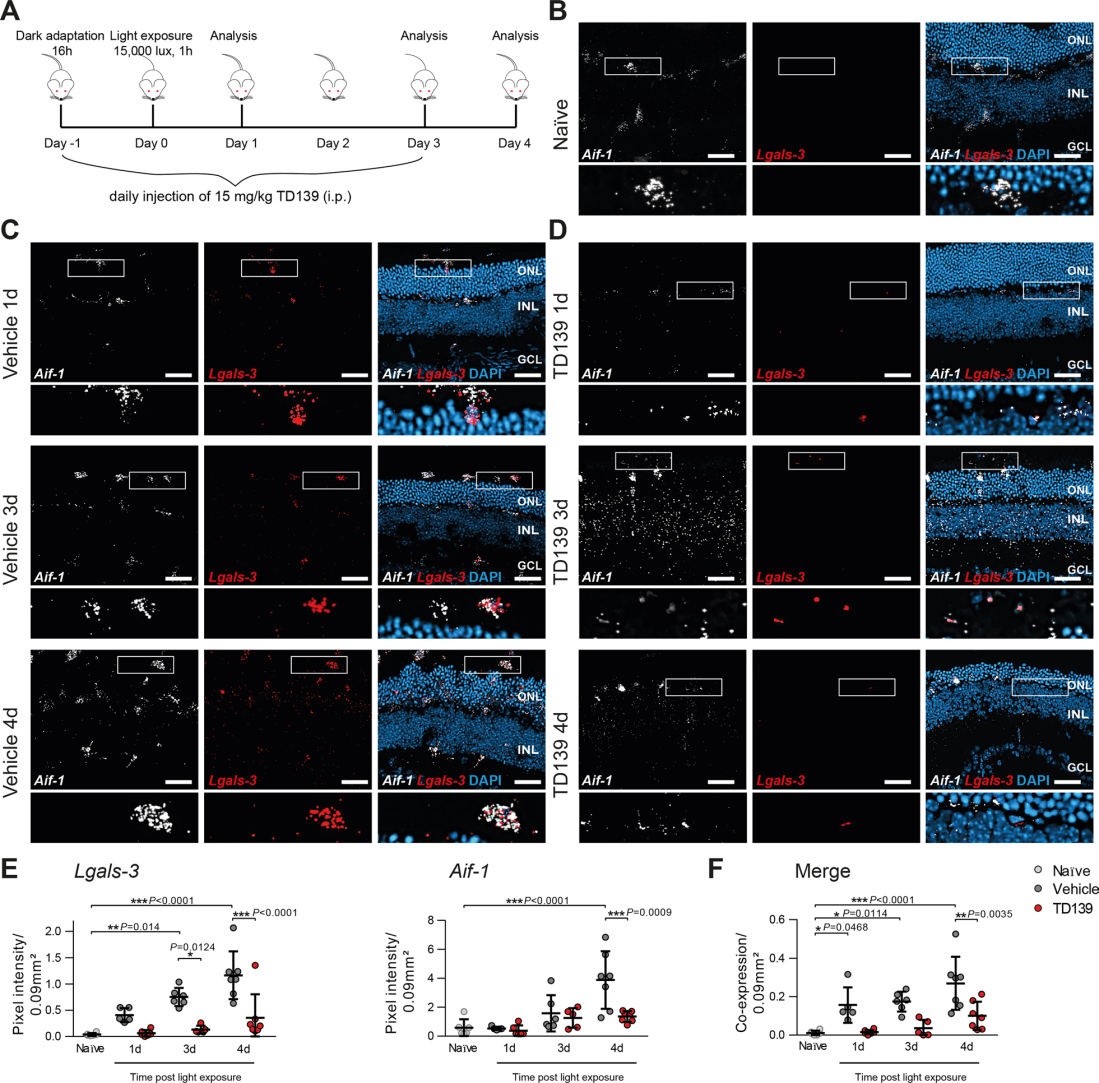
TD139 reduces *Lgals-3* expression in light-exposed retinas. **A** Light exposure regimen of BALB/cJ mice and TD139 administration. **B** Representative images of in situ hybridization of retinal cross-sections from naïve BALB/cJ mice using mRNA probes for *Aif-1* to label microglia/macrophages in combination with *Lgals-3*. Inlays show higher magnification. Scale bar: 50 µm. ONL, outer nuclear layer and INL, inner nuclear layer. **C**,** D** Representative images of in situ hybridization of retinal cross-sections of light-exposed mice treated with vehicle (**C**) or TD139 (**D**). Inlays show higher magnification. Scale bar: 50 µm. **E**,** F** Analysis of in situ hybridization signals of *Aif-1* and *Lgals*-*3* (**E**) and their merged co-expression (**F**) in light-exposed retinas at the indicated time points using pixel intensities within a defined area of 0.09 mm^2^ from the entire retina. Data show mean ± SEM. Naïve *n* = 6; Vehicle/TD139: 1d *n* = 5/6; 3d *n* = 6/5; 4d *n* = 7 retinal cross-sections. *P < 0.05 and ***P ≤ 0.001 by ordinary one-way ANOVA followed by Tukey’s multiple comparisons

To validate the findings from genetic knockout with a translational therapy approach, we studied whether pharmacological inhibition of galectin-3 effectively attenuates microgliosis and retinal degeneration. We performed light exposure of BALB/cJ mice that were treated daily with the small-molecule galectin-3 inhibitor TD139 or vehicle control (Fig. 5A). TD139, a thiodigalactoside derivate with high binding affinity for the galectin-3 carbohydrate recognition domain, has previously shown efficacy in murine models of lung fibrosis and corneal neovascularization [32, 33]. We first analyzed the effect of TD139 on galectin-3 expression itself using simultaneous in situ hybridization of retinal cross-sections with probes targeting *Aif-1* (Iba-1) and *Lgals-3* (Fig. 5B–D). The specificity of the mRNA probe *Aif-1* for labeling microglia was validated by co-immunofluorescence with Iba-1 (Additional file 1: Fig. S1). In situ hybridization of galectin-3 in retinal cross-sections showed that microglia in naïve mice express only low levels of *Lgals-3* (Fig. 5B, E, F), whereas increased expression levels were detected in activated *Aif-1*^+^ microglia in the ONL and subretinal space after light exposure in vehicle-treated mice (Fig. 5C, E, F). Mice that received TD139 showed significantly reduced *Lgals-3* expression in microglia (Fig. 5D-F). To confirm these in situ data, we also analyzed transcript levels of *Lgals*-3 in light-exposed retinas and RPE/choroids using qPCR. Here, we found increased galectin-3 mRNA levels after light exposure, which were reduced after TD139 treatment in both retinal and RPE/choroidal samples (Additional file 1: Fig. S2A, B). These data indicate that TD139 markedly reduces galectin-3 expression associated with microglia in light-exposed mouse retinas. Interestingly, co-staining for *Lgals-3* with CD45, a marker that can distinguish infiltrating myeloid cells from resident microglia, showed the presence of CD45^+^ immigrant cells after light-damage, but these cells displayed no specific galectin-3 expression (Additional file 1: Fig. S3). This indicates that damage-associated galectin-3 expression is predominantly present in resident myeloid cells.

**Fig. 6:**
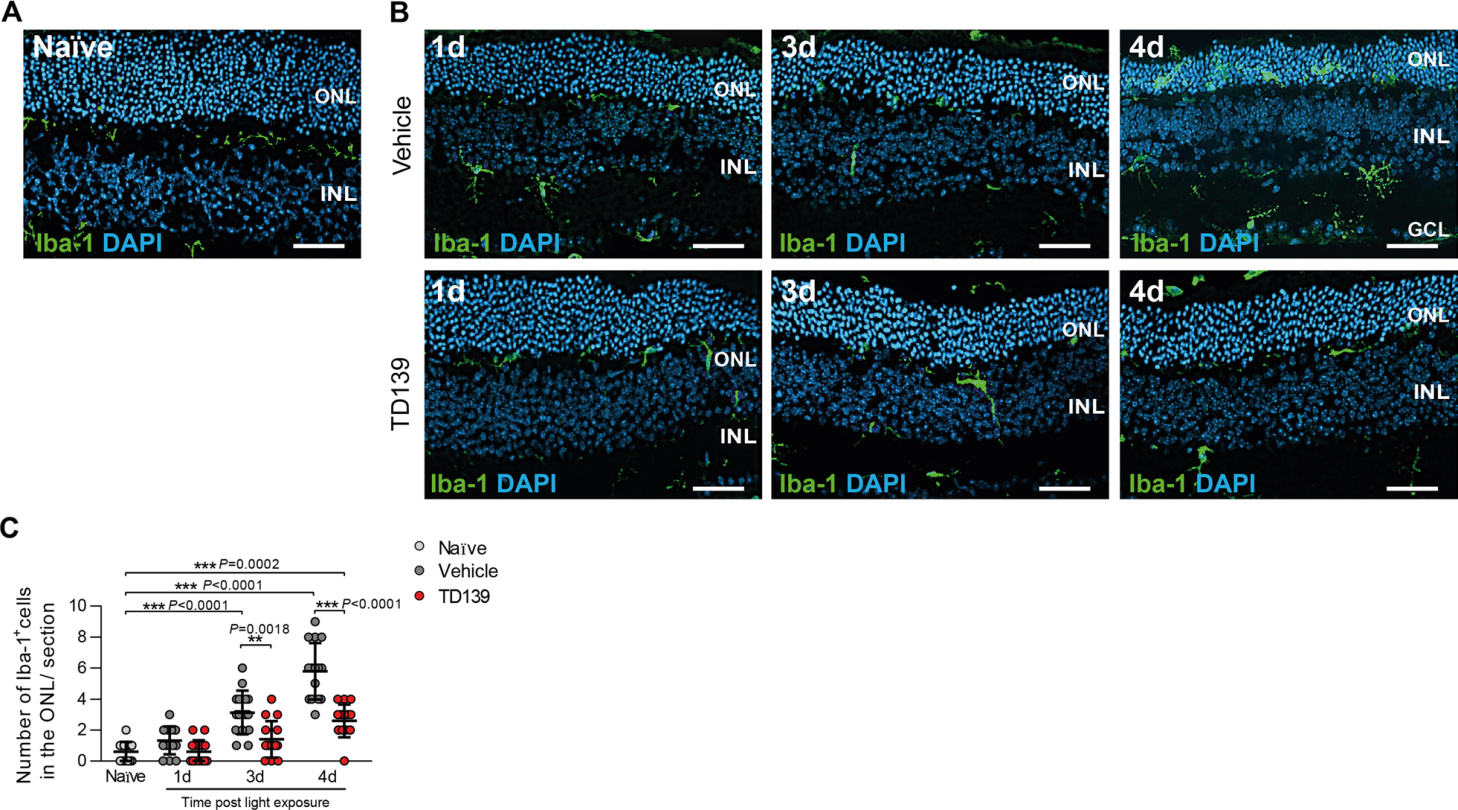
TD139 reduces microglia migration in light-exposed retinas. Representative images of Iba-1^+^ cells in naïve (**A**) and light-exposed mice treated with vehicle or TD139 (**B**) at the indicated time points. Scale bar: 50 µm. ONL, outer nuclear layer and INL, inner nuclear layer. **C** Quantification of Iba-1^+^ cells in the ONL. Data show mean ± SEM. *n* = 15 retinas from individual mice. **P < 0.01 and ***P ≤ 0.001 by ordinary one-way ANOVA followed by Tukey’s multiple comparisons

We next determined if blocking of galectin-3 by TD139 also modulates microglia morphology, migration and inflammatory gene expression. TD139-treated mice showed less microglia migration and accumulation of activated immune cells in the ONL and subretinal space at day 3 and 4 post-light exposure compared to vehicle-treated mice (Fig. 6A–C). Of note, four days post-light exposure, TD139-treated mice still showed higher numbers of microglia in the ONL compared to naïve controls, indicating that galectin-3 inhibition could not completely block microglia activation. In these stainings, we also noted that thinning of the ONL was less in conditions of TD139 treatment (Fig. 6B).

**Fig. 7:**
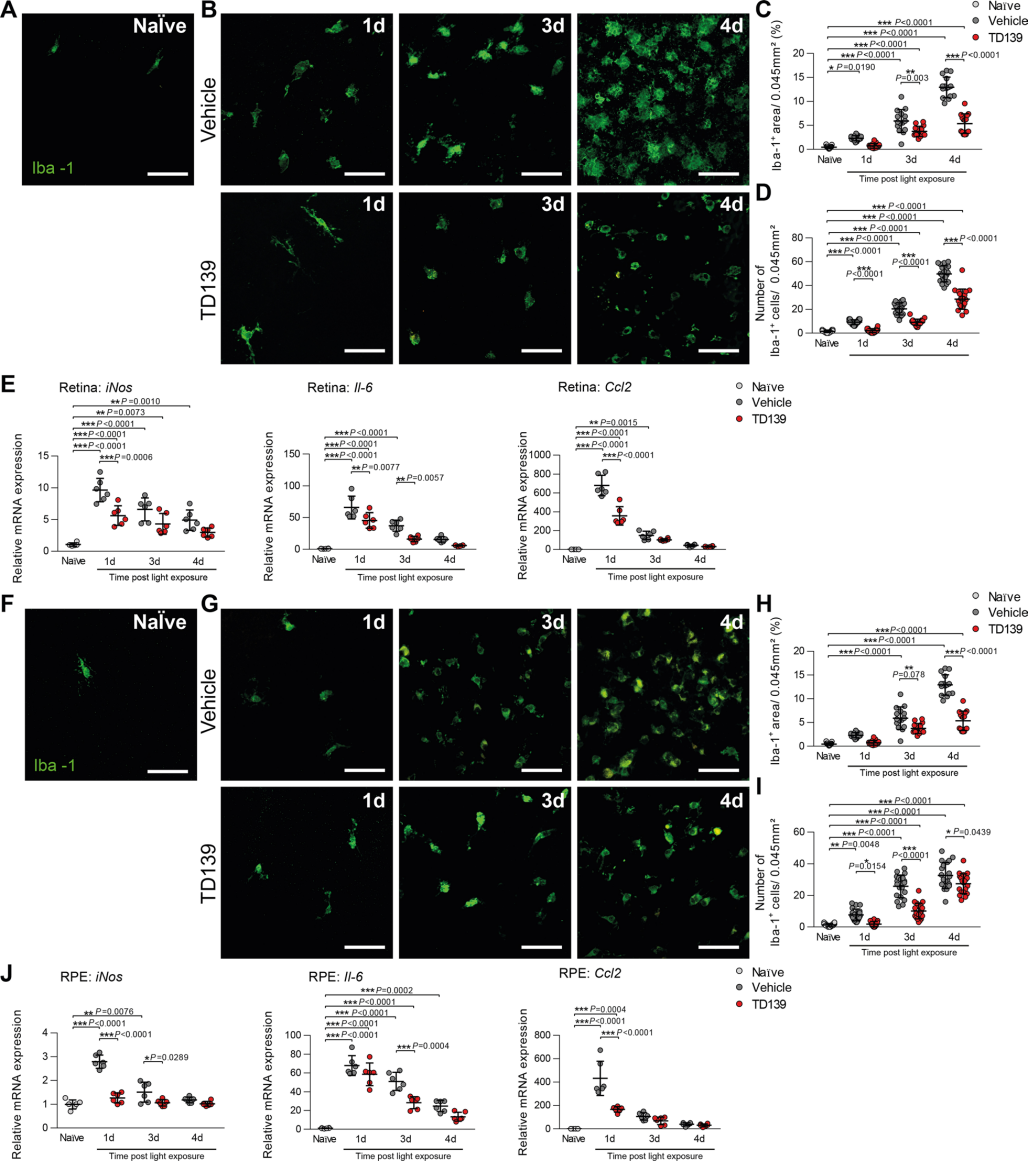
TD139 dampens microglia reactivity in light-exposed mice. Representative images of Iba-1^+^ cells in the subretinal space of retinal flat mounts from naïve (**A**) and light-exposed mice treated with vehicle or TD139 (**B**). Scale bar: 50 µm. **C** Quantification of Iba-1^+^ area in the subretinal space. *n* = 15 retinas from individual mice. **D** Number of Iba-1^+^ cells in the subretinal space. *n* = 15 retinas from individual mice. **E** Relative mRNA expression of pro-inflammatory cytokines in retinas of naïve and light-exposed mice treated with vehicle or TD139 normalized to the reference gene Atp5b. *n* = 6 retinas from individual mice. Representative images of Iba-1^+^ cells in RPE/choroids from naïve (**F**) and light-exposed mice treated with vehicle or TD139 (**G**). Scale bar: 50 µm. **H** Quantification of Iba-1^+^ area RPE/choroidal flat mounts. *n* = 15 RPE/choroids from individual mice. **I** Number of Iba-1^+^ cells in the RPE/choroid. *n* = 15 RPE/choroids from individual mice. **J** Relative mRNA expression of pro-inflammatory cytokines in RPE/choroids of naïve and light-exposed mice treated with vehicle or TD139 normalized to the reference gene Atp5b. *n* = 6 RPE/choroids from individual mice. Data show mean ± SEM. *P < 0.05; **P < 0.01 and ***P ≤ 0.001 by ordinary one-way ANOVA followed by Tukey’s multiple comparisons

The following flat-mount analysis of the retina showed that less amoeboid and more ramified microglia were detected in the subretinal space of TD139-treated mice after light exposure compared to the vehicle group (Fig. 7A–D). Light damage-induced expression of *iNos*, *Il-6*, Ccl2 was also reduced after TD139 treatment (Fig. 7E). Similar to the retina, the analysis of RPE/choroidal flat mounts showed reduced microglia accumulation and activation in light-exposed mice after TD139 treatment (Fig. 7F–J). Collectively, these data indicate that inhibition of galectin-3 via TD139 potently reduces retinal microglia reactivity.

**Fig. 8:**
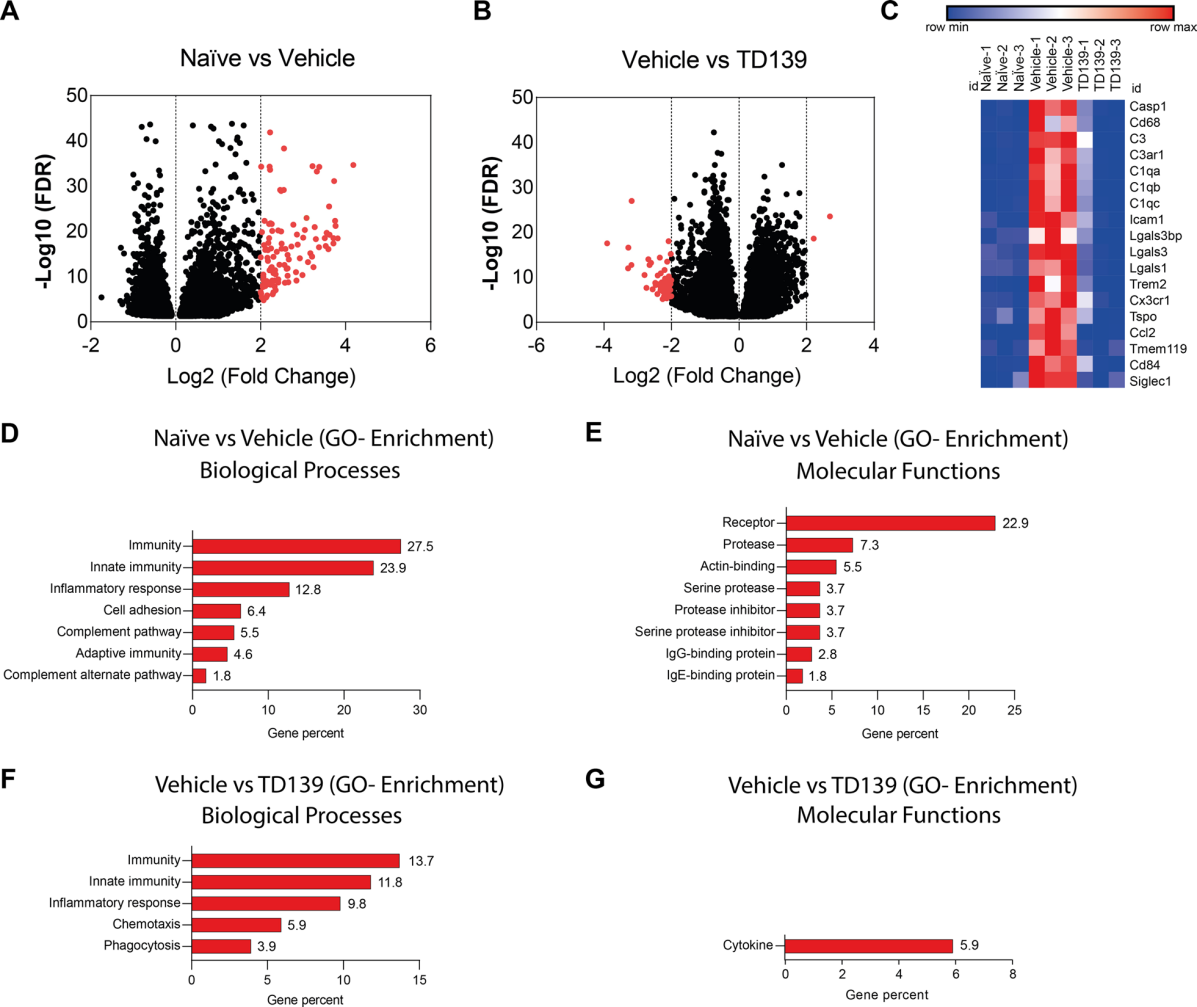
Global transcriptomic changes of retinas from naïve and light-exposed mice treated with vehicle or TD139. Volcano plots derived from RNA-sequencing data showing the comparison of differentially expressed genes (DEGs) from naïve mice versus vehicle-treated mice (**A**) and the comparison between light-exposed mice treated with vehicle versus TD139 (**B**). **C** Heat map showing the expression changes of selected DEGs in retinas from naïve animals and light-exposed mice treated with vehicle or TD139. Gene ontology (GO) enrichment analysis (biological processes and molecular functions) of DEGs from naïve mice versus vehicle-treated mice (**D**,** E**) and DEGs from light-exposed mice treated with vehicle versus TD139 (**F**,** G**). Relative gene percentage is indicated for each bar. *n* = 3 biological replicates using retinas from individual mice

To further analyze the immune-related transcriptomic changes elicited by galectin-3 inhibition in the retina, RNA-sequencing was performed in biological triplicates of naïve and light-exposed animals treated with TD139 or vehicle (Fig. 8). A very stringent cut-off was applied, where only transcripts with a log2-fold change of ≥ 2 or ≤  − 2 were considered as differentially expressed. We first compared expression levels of naïve versus vehicle-treated light-damage mice (Fig. 8A) and found that all differentially expressed genes (DEGs) were upregulated (114 DEGs up versus 0 DEGs down), including *Lgals-3* and *Ccl2*, corroborating our previous data (Fig. 5). Conversely, the comparison of vehicle- versus TD139-treated light-damage mice (Fig. 8B) detected many downregulated genes in the galectin-3 inhibition group (2 DEGs up versus 51 DEGs down). These findings indicate that most immune-related gene expression in the light-damaged retina is potently reduced by TD139 therapy. This is also illustrated by a heat map of selected DEGs representing macrophage activation (*Cd68, Tspo, Ccl2, Casp1*), complement factors (*C3*) and receptors (*C3ar1*, *C1qa-c*), integrins (*Icam1*), lectins (*Lgals-1*, *Lgals-3*, *Lgals-3 bp, Siglec-1*), and microglia signature genes (*Cx3cr1*, *Tmem119*) (Fig. 8C). Gene ontology enrichment analysis also revealed that the over-represented biological processes and molecular functions in the light-damaged vehicle-treated retina are related to immunity (Fig. 8D) and receptors (Fig. 8E), respectively. Likewise, TD139 treatment mainly affected GO-enrichment terms ′Immunity′ (GO biological process) (Fig. 8F) and ′Cytokine′ (GO molecular function) (Fig. 8G), respectively.

**Fig. 9:**
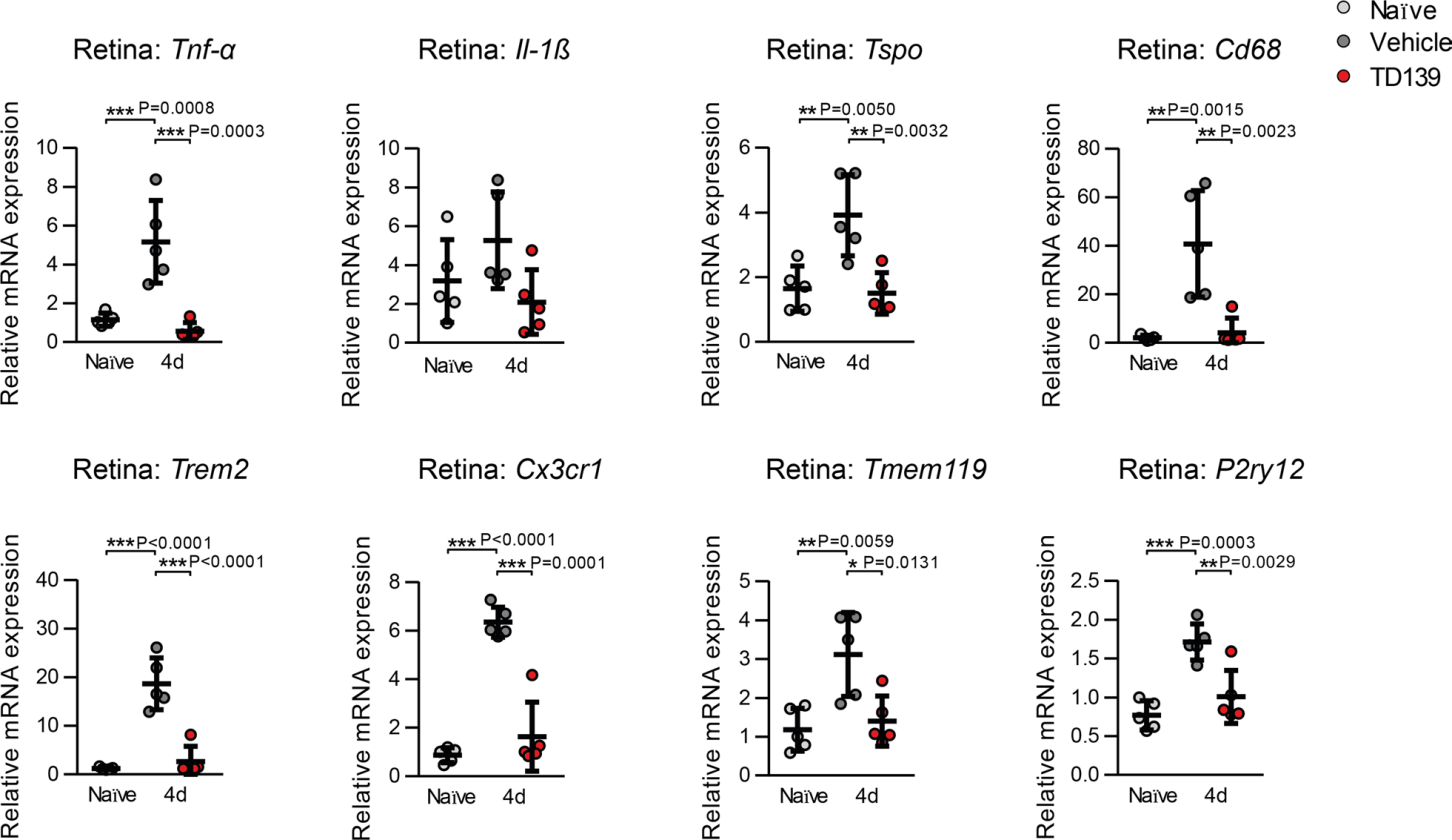
Validation of inflammatory markers and microglia signature genes in naïve versus light-exposed BALB/cJ mice. qRT-PCR analysis of relative mRNA expression in the retina 4 days post-light exposure of naïve and light-exposed mice treated with vehicle or TD139 normalized to the reference gene Atp5b. *n* = 5 retinas from individual mice. Data show mean ± SEM. *P < 0.05; **P < 0.01 and ***P ≤ 0.001 by ordinary one-way ANOVA followed by Tukey’s multiple comparisons

We next performed qRT-PCR validation of the transcriptomic data with a focus on microglia/macrophage activation markers (*Tnf-α*, *Il-1ß*, *Tspo*, *Cd68*) and signature genes (*Trem2, Cx3cr1*, *Trem119*, *P2ry12*). All analyzed transcripts were induced by light damage and this upregulation was significantly reduced in the TD139 treatment group of animals (Fig. 9). These findings clearly show that galectin-3 inhibition potently affects immune-related gene expression in the retina challenged with acute white light.

### TD139 reduces overt light-induced retinal degeneration

**Fig. 10:**
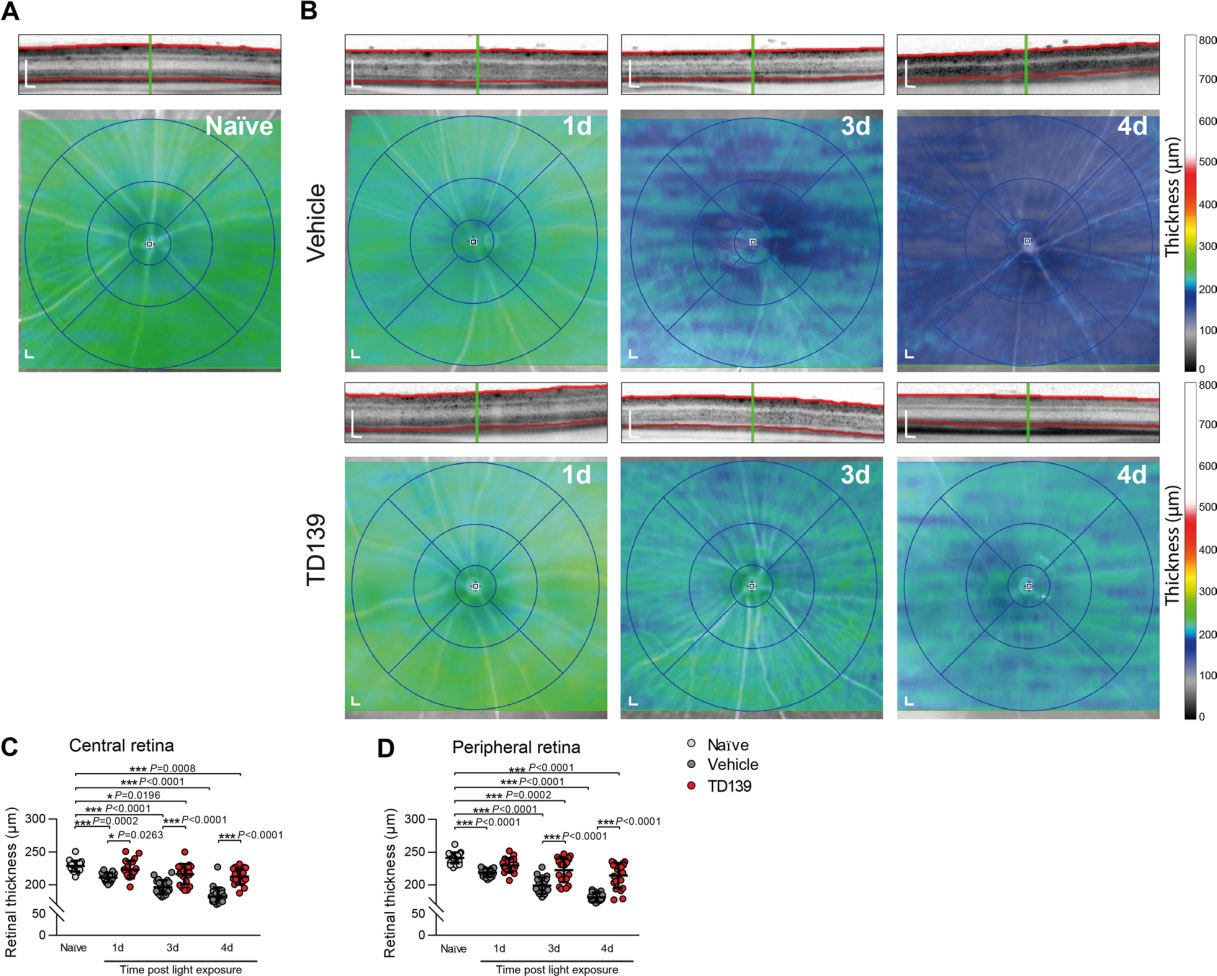
TD139 delays light-induced retinal degeneration. Representative SD-OCT scans and heat maps of retinal thickness from naïve (**A**) and light-exposed mice treated with vehicle or TD139 (**B**). Scale bar: 200 µm. Quantification of the averaged thickness of the central retina (circle diameter 3 mm) (**C**) and peripheral retina (circle diameter 6 mm) (**D**). Data show mean ± SEM. Naïve *n* = 20; Vehicle/TD139: 1d *n* = 19/20; 3d *n* = 20; 4d *n* = 19/20 eyes. *P < 0.05 and ***P ≤ 0.001 by ordinary one-way ANOVA followed by Tukey’s multiple comparisons

We then studied the structural effects of TD139 on light-induced retinal degeneration. SD-OCT scans of light-exposed vehicle-treated mice showed a prominent progressive retinal thinning, especially of the ONL, which was attenuated in TD139-treated mice. Here, the retinal thickness and structure were relatively preserved compared to the vehicle group (Fig. 10). Since TD139 has been reported to have a weak side effect potentially mediated by galectin-1 [34], we examined its specificity in galectin-3-deficient mice. Notably, TD139 treatment did not result in any neuroprotective effect in light-exposed *Lgals*-3 KO mice (Additional file 1: Fig. S4). These data clearly indicate that the protective effects of TD139 in the retina are predominantly mediated via specific inhibition of galectin-3.

### TD139 reduces focal blue light-induced retinal damage

**Fig. 11:**
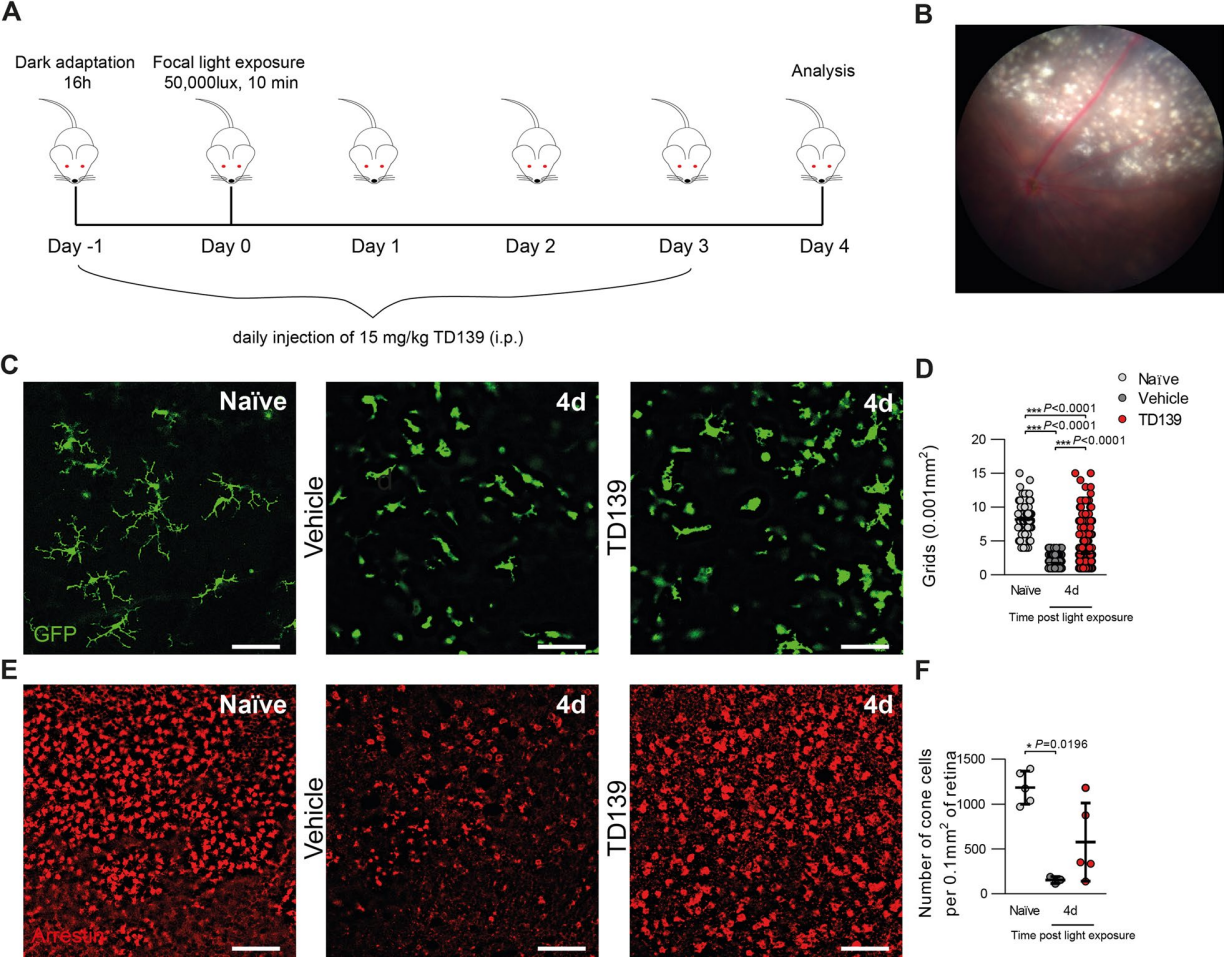
TD139 reduces microglia reactivity and delays cone photoreceptor death in focal light induced damage. **A** Light exposure regimen of focal blue light damage and TD139 administration in Cx3cr1^GFP/+^ reporter mice. **B** Representative fundus image of a focal light lesion of a vehicle-treated mouse 4 days post-light exposure. **C** Representative images of GFP^+^ microglia in the OPL of retinal flat mounts of naïve and light-exposed mice treated with vehicle or TD139. Scale bar: 50 µm. **D** Quantification of grid crossing points from GFP^+^ cells. Data show mean ± SEM. Naïve *n* = 128; Vehicle/TD139: 4d *n* = 309/428 cells. ***P ≤ 0.001 by ordinary one-way ANOVA followed by Tukey’s multiple comparisons. **E** Representative images of cone arrestin^+^ cells in retinal flat mounts of naïve and light-exposed mice treated with vehicle or TD139. Scale bar: 50 µm. **F** Quantification of cone arrestin^+^ cells in the retina. Data show mean ± SEM. Naïve *n* = 5; Vehicle/TD139: 4d *n* = 3/5 retinas. *P < 0.05 by Kruskal–Wallis test followed by Dunn’s multiple comparison

Finally, to further validate our findings in an independent retinal degeneration model, Cx3cr1^GFP/+^ reporter mice were exposed to 50,000 lux focal white light delivered by an otoscope for 10 min (Fig. 11A). Analysis of fundus images of vehicle-treated mice revealed a focal atrophic retinal damage (Fig. 11B). While focal light-damaged retinas showed a disturbed microglia network with less ramified and more amoeboid-shaped cells within the atrophic lesion, more ramified microglia were found in TD139-treated mice (Fig. 11C, D). We next assessed the effect of TD139 treatment on cone photoreceptor degeneration. Here, cone arrestin-stained retinal flat mounts showed a massive loss of cone photoreceptors in the lesion site, which was significantly less pronounced in TD139-treated mice (Fig. 11E, F). Thus, TD139 reduces microglia reactivity and ameliorates cone photoreceptor survival in an independent mouse model of focal light-induced retinal degeneration.

## Discussion

Aberrant immune responses, including reactive microgliosis, are hallmarks of neurodegenerative diseases. Galectin-3 has been defined as a mediator of microglial inflammatory responses in different preclinical models of brain degeneration [16, 23, 25, 28]. In this report, we define a novel role for galectin-3 in retinal degeneration and blinding diseases. Thus, we found increased galectin-3 expression in retinas of dry AMD patients and similarly in a translational mouse model using two different light-damage paradigms. Targeting of galectin-3 by genetic knockout or using the small-molecule inhibitor TD139 could limit microglia reactivity and reduce retinal damage in these two different mouse models of light-induced retinal degeneration.

Several studies reported increased galectin-3 levels during the onset and progression of disease pathology in neurodegenerative diseases [21, 23, 25, 28]. In the eye, a proteomic screening identified high galectin-3 levels as a biomarker in AMD patient specimens [31]. Consistent with this global proteomics approach, we found increased galectin-3 expression in retinal lesions of dry AMD patients and showed that activated microglia are one of the major sources.

Two recent studies demonstrated that galectin-3 expression was highly upregulated in activated microglia in lesion sites in brains of AD and HD patients which was also validated in the corresponding mouse models [21, 23]. Similar findings were documented by our data from light-exposed mice, which revealed defined galectin-3 expression in activated microglia.

Our RNA-seq analysis of naïve and light-damaged retinas clearly showed that *Lgals-3* transcripts were strongly induced by light exposure and that this induction could be prevented by TD139 treatment. These findings corroborate a recent concept, where *Lgals-3* has been identified as a marker in a single cell NGS cluster analysis as well as in in situ hybridization of subretinal microglia in light damage as well as rhodopsin mutant retinas [24]. In good agreement with our data, the group also identified downregulation of homeostatic microglia genes under light-damage conditions including *Cx3cr1*, *Trem119*, and *P2ry12* [24]. Our in situ analysis of AMD patient sections further showed that not only activated microglia, but also other cells in the subretinal space of human AMD patients could express galectin-3. The origin and phenotype of these cells in the damaged human retina deserves further attention.

Galectin-3 is a pleiotropic protein with diverse functions in physiological and pathophysiological conditions, including functions in cell proliferation, migration and innate immune response [35–37]. To determine if galectin-3 modulates microglia activity during light-induced retinal degeneration, we used galectin-3 (*Lgals*-3) KO mice. Our data demonstrate that microglia from *Lgals*-3 KO mice showed no morphological differences or other phenotypic abnormalities compared to WT littermates but much less reactivity. In line with this notion, a previous study using galectin-3-deficient primary adult microglia reported no overt phenotype under basal homeostatic conditions but a significant decrease in activation and proliferation in response to ischemic brain injury [16]. In another report, primary cultured rat microglia stimulated with recombinant galectin-3 showed increased expression of inflammatory mediators including Tnf-α, Il-6 and Il-1β [38]. Conversely, blocking galectin-3 with modified citrus pectin (MCP) attenuated the inflammatory response of stimulated BV-2 microglia cells [39]. Interestingly, several studies have shown that galectin-3 can initiate TLR4- and JAK/STAT-dependent inflammatory response in microglia [15, 16, 38].

Glycosylation plays a pivotal role in the innate and adaptive immune response. In fact, phagocytosis of dysfunctional neurons depends on desialylation of cell surface glycans and subsequent binding of galectin-3 which serves as an opsonizing factor [40]. In line with this, our data showed that galectin-3 deficiency has neuroprotective effects and preserves retinal structure in light-damaged mice. Moreover, other studies supporting a pro-inflammatory role for galectin-3 in the retina showed that loss of galectin-3 is associated with less severe retinal disease and neuroinflammation of the optic nerve tissue during chemically induced diabetes [28, 41]. However, in traumatic brain injury, both neuroprotective and neurodegenerative effects of galectin-3 deficiency have been described [16, 23, 42]. This observed discrepancy suggests a complex role for galectin-3 that differs depending on the disease severity and progression.

The prominent role of galectin-3 in physiological and pathological processes resulted in the development of several promising synthetic small-molecule galectin-3 inhibitors with both research and clinical implications. TD139, a 3,3’-bis-(4-aryltriazol-1-yl) thiodigalactoside has been considered as the most advanced small-molecule galectin-3 inhibitor and is now in clinical trials for idiopathic pulmonary fibrosis (IPF) [33]. Previous studies have shown therapeutic effects of TD139 in mouse models of IPF [43, 44], chemical injury-induced pathological corneal neovascularization and fibrosis [32] as well as in experimental autoimmune uveitis (EAU) [45]. Here, using two different experimental mouse models, we showed promising effects of TD139 treatment in delaying microgliosis and attenuating retinal degeneration.

Notably, the selectivity of TD139 for galectin-3 has been challenged as cross-reactivity with galectin-1 was demonstrated [34, 46, 47]. We cannot exclude the possibility that TD139 also targeted galectin-1 in our retinal models. However, as galectin-3-deficient mice treated with TD139 did not show any neuroprotective effect in light-induced retinal degeneration, we conclude that the therapeutic effects are mainly mediated via inhibition of galectin-3.

## Conclusion

Our findings demonstrate that increased galectin-3 in microglia contributes to inflammation and disease progression, while targeting galectin-3 by genetic or pharmacological approaches ameliorates light-induced retinal degeneration. These findings highlight a potential new therapeutic strategy for treating retinal inflammatory diseases such as AMD.

## Supplementary Information

**Additional file 1:** Supplementary Figures 1–4.

## Data Availability

The data supporting the findings of this study are available from the corresponding author upon request. The RNA-seq raw data and normalized DESeq2 counts of this study are publicly available at the National Center for Biotechnology Information Gene Expression Omnibus (http://www.ncbi.nlm.nih.gov/geo/) as series record GSE211119.

## Abbreviations

AD: Alzheimer′s disease
*Aif-1*: Allograft inflammatory factor-1
AMD: Age-related macular degeneration
*Ccl2*: C–C motif chemokine ligand 2
Cx3cr1: CX3C chemokine receptor 1
DEGs: Differentially expressed genes
EAU: Experimental autoimmune uveitis
GA: Geographic atrophy
HD: Huntington’s disease
Iba-1: Ionized calcium-binding adapter molecule 1
iNos: Inducible NO synthase
IL-1ß: Interleukin-1ß
IL-6: Interleukin-6
IPF: Idiopathic pulmonary fibrosis
IPL: Inner plexiform layer
ONL: Outer nuclear layer
OPL: Outer plexiform layer
PD: Parkinson′s disease
P2yr12: Purinergic receptor P2Y12
RPE: Retinal pigment epithelium
SD-OCT: Spectral domain optical coherence tomography
TLR: Toll-like receptor
TMEM: Transmembrane protein
TNF: Tumor necrosis factor
Trem2: Triggering receptor expressed on myeloid cells 2
TSPO: Translocator protein 18 kDa
VEGF: Vascular endothelial growth factor
