# Supplementary material for: Genetic targeting or pharmacological inhibition of galectin-3 dampens microglia reactivity and delays retinal degeneration
Source: J Neuroinflammation. 2022 Sep 17;19:229. doi: 10.1186/s12974-022-02589-6 (PMC9482176; doi:10.1186/s12974-022-02589-6)
Supplement: Supplementary file 1 — Additional file 1: Supplementary Figures 1–4. [file 12974_2022_2589_MOESM1_ESM.docx]

Supplementary information

**Genetic targeting or pharmacological inhibition of galectin-3 dampens microglia reactivity and delays retinal degeneration**

**Mona Tabel, Anne Wolf, Manon Szczepan, Heping Xu, Herbert Jägle, Christoph Moehle, Mei Chen and Thomas Langmann**

Supplementary Figures 1-4

**Supplementary Figure 1.**


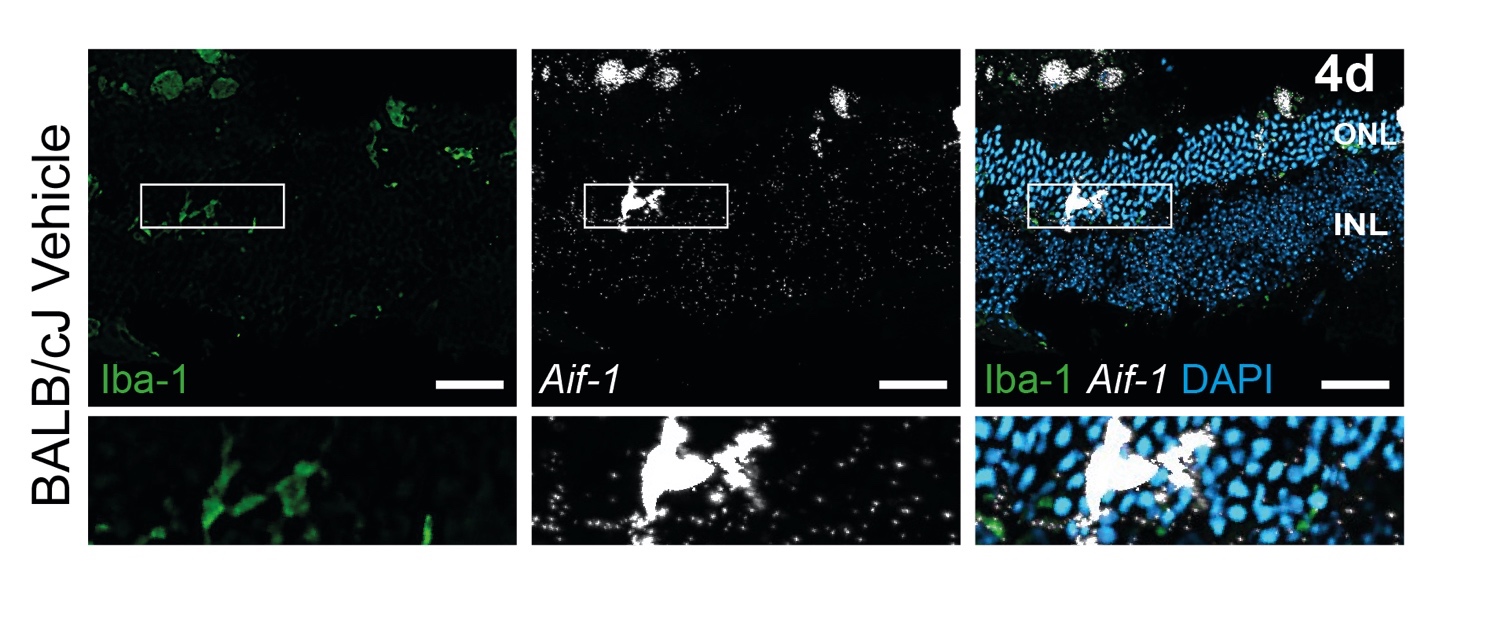


**Supplementary Figure 1: *Aif-1* mRNA probe labels retinal microglia.**

Representative images of immunofluorescence *in situ* hybridization of retinal cross-sections of light-exposed BALB/cJ mice stained with Iba-1 and mRNA probe for *Aif-1*. Inlays show higher magnification. Scale bar: 50 µm. ONL, outer nuclear layer and INL, inner nuclear layer**.**

**Supplementary Figure 2.**


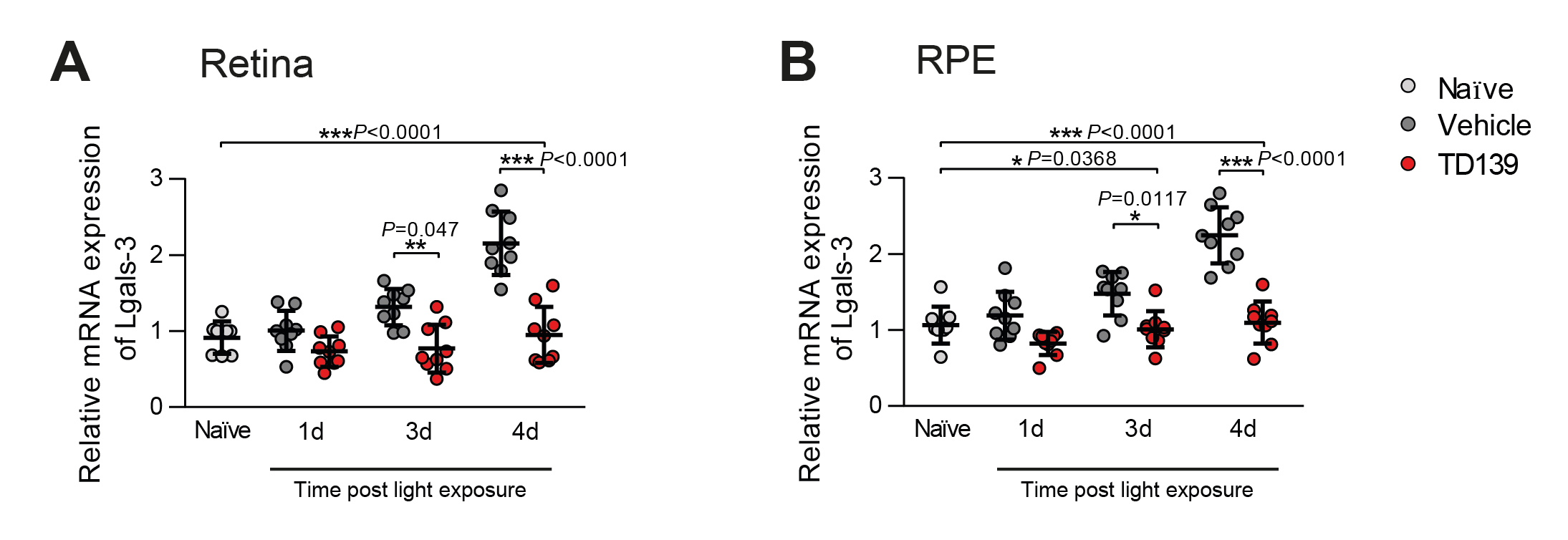


**Supplementary Figure 2: Increased galectin-3 expression in light-exposed BALB/cJ mice.**

**A-B** Galectin-3 transcript levels in the retina **(A)** and RPE/choroid **(B)** of naïve and light-exposed mice treated with vehicle or TD139 calibrated to naïve tissue. Data show mean ± SEM. naïve n= 8/9; 1d n= 9; 3d n= 9; 4d n= 9 retinas or RPE/choroids. **P* < 0.05; ***P* < 0.01 and ****P* ≤ 0.001 by ordinary one-way ANOVA followed by Tukey’s multiple comparisons.

**Supplementary Figure 3.**

**
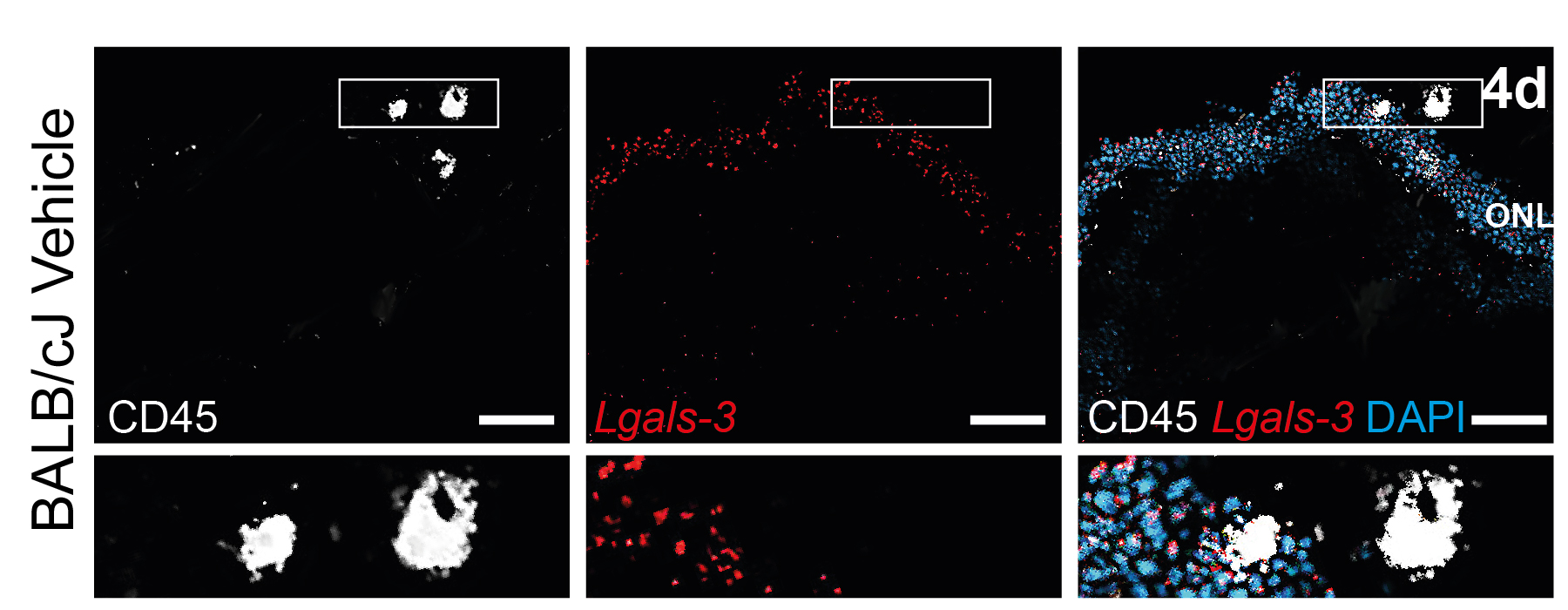
**

**Supplementary Figure 3: Lgals-3 expression and infiltrating monocytes in light exposed retina.**

**A** Immunofluorescence *in situ* hybridization of retinal cross-sections of light-exposed BALB/cJ mice stained with anti-CD45 antibody and mRNA probe for *Lgals-3*. Inlays show higher magnification. Scale bar: 50mm. ONL, outer nuclear layer and INL, inner nuclear layer**.**

**Supplementary Figure 4.**

**
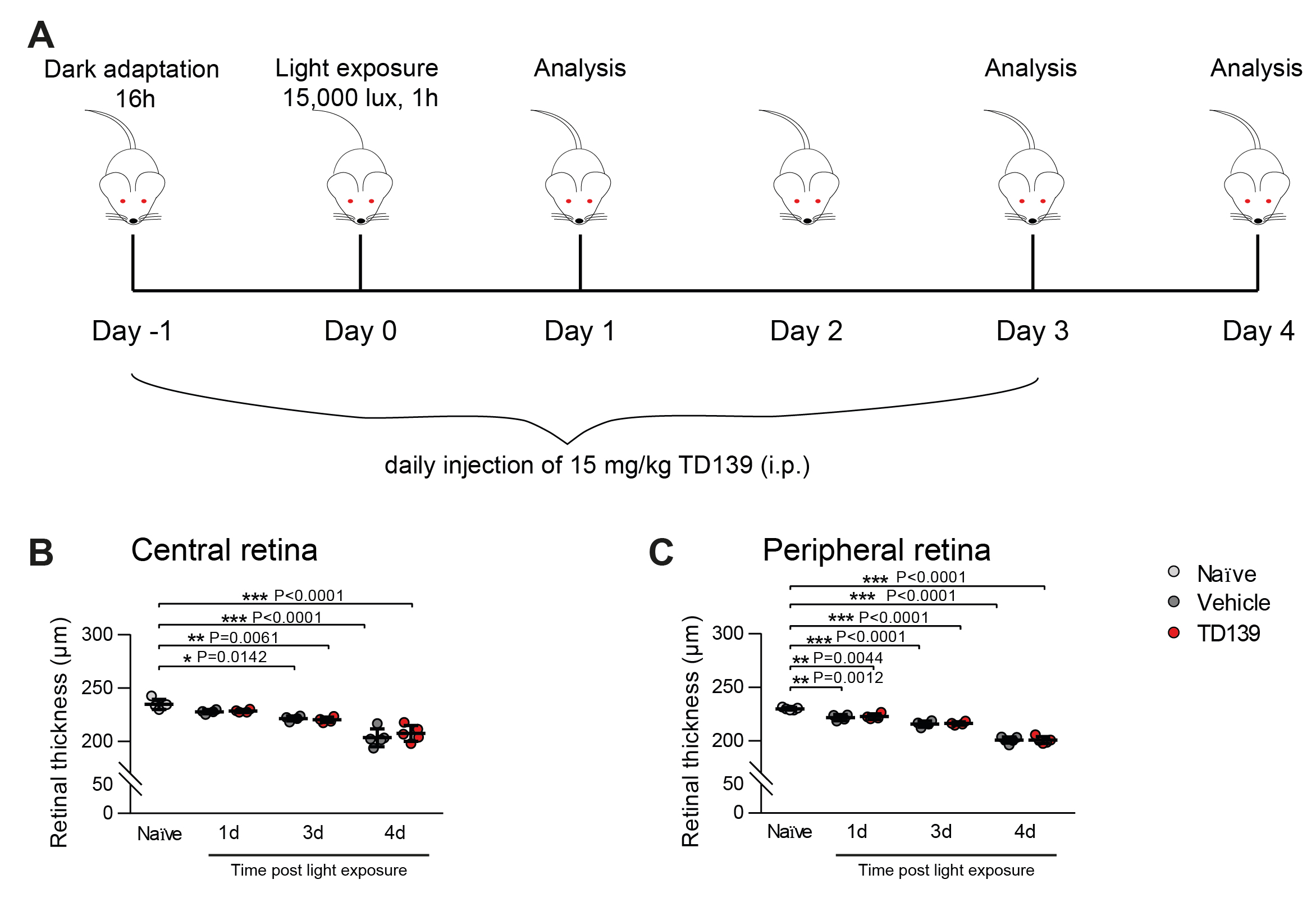
**

**Supplementary Figure 4: Effect of TD139 treatment on light-exposed galectin-3-deficient mice**.

**A** Light exposure regimen of *Lgals-3* KO mice treated with vehicle or TD139. **B-C** Quantification of the average thickness of the central retina (circle diameter 3 mm) **(B)** and peripheral retina (circle diameter 6 mm) **(C)** of *Lgals-3* KO mice. Data show mean ± SEM. Vehicle/TD139 naïve n= 5; 1d n= 4; 3d n= 4; 4d n= 5 eyes. **P* < 0.05; ***P* < 0.01 and ****P* ≤ 0.001 by ordinary one-way ANOVA followed by Tukey’s multiple comparisons.
